# Hydrological dataset of a sub-humid continental plain basin (Buenos Aires, Argentina)

**DOI:** 10.1016/j.dib.2020.106400

**Published:** 2020-10-10

**Authors:** María Emilia Zabala, Ricardo Sánchez Murillo, Sebastián Dietrich, Martín Gorocito, Luis Vives, Marisol Manzano, Marcelo Varni

**Affiliations:** a Instituto de Hidrología de Llanuras “Dr. Eduardo Jorge Usunoff” (IHLLA), República de Italia 780, Azul, Buenos Aires, Argentina; b Consejo Nacional de Investigaciones Científicas y Técnicas (CONICET), *Av*. Rivadavia 1917, Ciudad Autónoma de Buenos Aires, Argentina; c Universidad Nacional del Centro de la Provincia de Buenos Aires, Pinto 399, Tandil, Buenos Aires, Argentina; d Stable Isotopes Research Group and Water Resources Management Laboratory, Universidad Nacional, Heredia 86-3000, Costa Rica; e Agencia Nacional de Promoción Científica y Tecnológica, Godoy Cruz 2370, Ciudad Autónoma de Buenos Aires, Argentina; f Departamento de Ingeniería Minera y Civil, Universidad Politécnica de Cartagena, P° de Alfonso XIII 52, E-30203 Cartagena, España

**Keywords:** Argentina, Chaco-Pampean Plain, Water quantity and quality, Environmental tracers, Arsenic, Fluoride, Groundwater resources management

## Abstract

The Chaco-Pampean Plain (Argentina) is the strongest economic region and the most inhabited in the country, comprising approximately 66% of the country's population (26,500 million) [1]. In this region, surface slopes are very low (<0.1%) and due to the current climatological features, floods and droughts alternate over time. Salinity and alkalinity of water and soil increase towards the flattest sector of the basin, as well as the contents of arsenic and fluoride, which restrict their human use. Worldwide, population growth and global warming, in addition to political decisions, are leading to abrupt land use changes. Under this premise, identifying and quantifying the hydrological processes that control water quantity and its chemical quality become an imperative task [2]. This data article provides a long-term hydrological dataset from a sector of the Chaco-Pampean Plain, the Del Azul creek basin. Hydrological data such as flow rates and piezometric levels, and physical–chemical (i.e., major and minor solutes, and trace elements) and isotopic (δ^18^O, δ^2^H; and *d*-excess) data from rainwater, surface (creek and wetland) and groundwater (at two depths) are available. Rainwater samples are derived from three precipitation collectors installed at different altitudes (monitoring period: 2010–2019; *n* = 57). Surface water samples were collected at three sampling sites located along the Del Azul Creek and six wetlands (monitoring period: 2018–2019; *n* = 12). Groundwater samples were collected from 17 piezometers with depths ranging between 3 and 10 m, and from 12 piezometers of 30 m depth, all located throughout the entire basin (monitoring period: 2018–2019; *n* = 115). Sampling campaigns were performed during the austral dry (summer) and wet (spring) seasons. This dataset provides useful information to understand a) how water moves from recharge to discharge areas, b) how water acquires salinity, and c) how particular solutes of concern, such as arsenic and fluoride, are distributed in space and time across in an extensive plain.

## Specifications Table

**Table:** 

Subject	Earth-Surface Processes
Specific subject area	Chemical and stable isotopic characteristics of rainwater, surface water and groundwater from a large sub-humid plain.
Type of data	Table Image Graph
How data were acquired	Monthly rainfall amounts were recorded using automated weather stations (https://ihlla.conicet.gov.ar/bdh/) with remote data transmission (La Germania-LG) and manually (La Madrugada-LM). Rainfall amounts from the Instituto de Hidrología de Llanuras “Dr. Eduardo Jorge Usunoff” (IHLLA) station were provided by the Argentina National Weather Service (https://www.smn.gob.ar/). The superficial flow rates were measured with an OTT MF pro-Water Flow Meter equipment (Site 1) or calculated by rating curves at control sections (Sites 2 and 3). The phreatic level was measured using a piezometric probe. Chemical analyses were conducted at the IHLLA Laboratory (https://ihlla.conicet.gov.ar/laboratorio-de-analisis-quimicos/) following the methodology proposed by the American Public Health Association [3], and using the following methods: Ca^2+^, Mg^2+^, Na^+^ and K^+^ were analyzed by flame atomic absorption spectrometer (EAA SHIMADZU AA6800) with SM 3111 method; SO_4_^2−^ by ultraviolet spectrophotometer screening (Thermo Aquamate) with EPA 9038 method; F^−^ by ion-selective electrode (Orion model 720A) with SM 4500 D method; As by atomic absorption spectrometer - hydride generation (EAA SHIMADZU AA6800) with SM 3114 C method; Cl^−^ by titration with SM 4500 B method; NO_3_^−^ by ultraviolet spectrophotometer screening (Thermo Aquamate) with SM 4500 B-C method; silica (SiO_2_) by Ultraviolet spectrophotometer screening (Thermo Aquamate) with SM 4500 C method; CO_3_^−^ and HCO_3_^−^ (expressed as alkalinity) by titration with SM 2320 B method; electrical conductivity (EC) by a conductivity cell and pH by a potentiometric electrode (WTW Multi 9420). In all analyses charge-balance errors were <10%.
	Data on stable isotopes in rainwater are from different projects and were measured in four labs: the Geochronology and Isotope Geology Institute of Argentina (INGEIS in Spanish) [4][5]; the Geochronology Laboratory, Universidad de Salamanca (Spain) and the Water Laboratory of the Groundwater Hydrology Center, Universidad de Málaga (Spain), by mass spectrometry under continuous flow; and the Isotope Hydrology Laboratory of the Institute of Quaternary and Coast Geology, Universidad Nacional de Mar del Plata (Argentina), by Laser spectroscopy [6]. The analytical errors vary between ± 0.1‰ and ± 0.2‰ for δ^18^O, and between ± 1.0‰ and ± 2.0‰ for δ^2^H. Creek samples from 2018 were also analyzed at the Isotope Hydrology Laboratory of the Institute of Quaternary and Coast Geology. Wetlands and groundwater samples from 2019 were analyzed at the Stable Isotopes Research Group, Universidad Nacional (Heredia, Costa Rica). Stable isotopes analysis was conducted using a water isotope analyzer LWIA-45P (Los Gatos Research Inc., USA). The analytical long-term uncertainty was: ± 0.5 (‰) (1σ) for δ2H and ± 0.1 (‰) (1σ) for δ^18^O. Stable isotopes compositions are presented in delta notation δ (‰, per mil), relating the ratios (R) of ^18^O/^16^O and ^2^H/^1^H, relative to Vienna Standard Mean Ocean Water (V-SMOW). Deuterium excess was calculated as d-excess = δ^2^H-8•δ^18^O.
Data format	Raw
Parameters for data collection	All sampling sites are distributed throughout the basin, encompassing the upper, middle and lower basin in order to analyze the chemical spatial distribution. The rainwater sampling was performed taking into account how the rainfalls are seasonally distributed. Surface water and groundwater samplings were performed in two opposite hydrological situations. In the case of groundwater, the sampling was carried out at two depths (3–10 m and 30 m depth).
Description of data collection	Rainwater samples were collected using three integrating wet/dry precipitation collectors. Samples were collected on monthly basis and analyzed semi-annually as composite samples. Surface water samples were collected from three sites along the Del Azul Creek and from six wetlands. Groundwater samples were collected from 17 shallow piezometers and 12 deep piezometers after purging at least three times the borehole water volume and after EC, pH and water temperature were stabilized.
Data source location	Institution: Instituto de Hidrología de Llanuras “Dr. Eduardo Jorge Usunoff” (IHLLA, https://ihlla.conicet.gov.ar/) City/Town/Region: Azul, Buenos Aires Country: Argentina Latitude and longitude for collected samples/data: 36° 46′ 29.72″S – 59° 51′ 13.92″W
Data accessibility	With the articles and at Mendeley Data http://dx.doi.org/10.17632/b34kg4jx7r.1[7]
Related research article	M.E. Zabala, M. Manzano, L. Vives, The origin of groundwater composition in the Pampeano Aquifer underlying the Del Azul Creek basin, Argentina, Sci. Total Environ. 518–519 (2015) 168–188. https://doi.org/10.1016/j.scitotenv.2015.02.065 [8] M.E. Zabala, M. Gorocito, S. Dietrich, M. Varni, R. Sánchez Murillo, M. Manzano, E. Ceballos, Key hydrological processes in the Del Azul Creek basin, sub-humid Pampean, Sci. Total Environ. 2020. https://doi.org/10.1016/j.scitotenv.2020.142258[2]

## Value of the Data

• We provide hydrological and chemical data to characterize the key hydrological processes governing water quantity and quality in a large sub-humid plain.
• In a large sub-humid plain, the agricultural productivity is greatly influenced by hydrological processes. Our data will allow the scientific community to understand and compare how water and solutes move across these unique environments, as well as enable better water and soil management decisions.
• Our hydrological dataset from a large sub-humid plain located in South America will enhance modeling comparison exercises with other large plains across the world in order to categorize (similarities and differences) and understand the key hydrological processes governing the water quantity and chemical quality.
• Our database offers an extensive series of chemical and isotopic rainwater data from a large sub-humid plain.
• In a large sub-humid plain, the increase of salinity and alkalinity towards the flattest area is a relevant topic of environmental concern. Our dataset provides data of various chemical and isotopic analyses from rainwater, surface water and groundwater in order to analyze their spatial and temporal distribution.
• In alkaline environments, both arsenic and fluoride are released into groundwater. At high concentrations, both elements can limit the water use [9]. Our dataset provides contents of arsenic and fluoride to evaluate their behavior in this unique environment.

## 1 Data Description

**Fig. 1:**
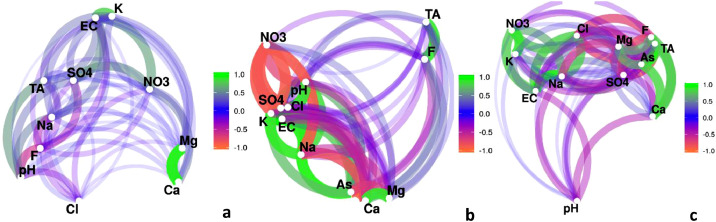
Pearson's correlation coefficient network diagrams for (a) precipitation, (b) creek and (c) wetlands. Precipitation samples (*n* = 57) were recorded from 2010 to 2019, creek samples (*n* = 6) were recorded in 2018 (summer and spring seasons) and wetlands samples (*n* = 6) were recorded in 2019 (spring season).

Fig. 1 shows the Pearson's correlation coefficient network diagram performed with data from three precipitation stations located in the upper (221 masl), middle (145 masl) and lower (72 masl) basin (a) [2,8], data from three sampling sites located along the Del Azul Creek (b) and with data from six wetlands located along a SW-NE flow line (c).

**Fig. 2:**
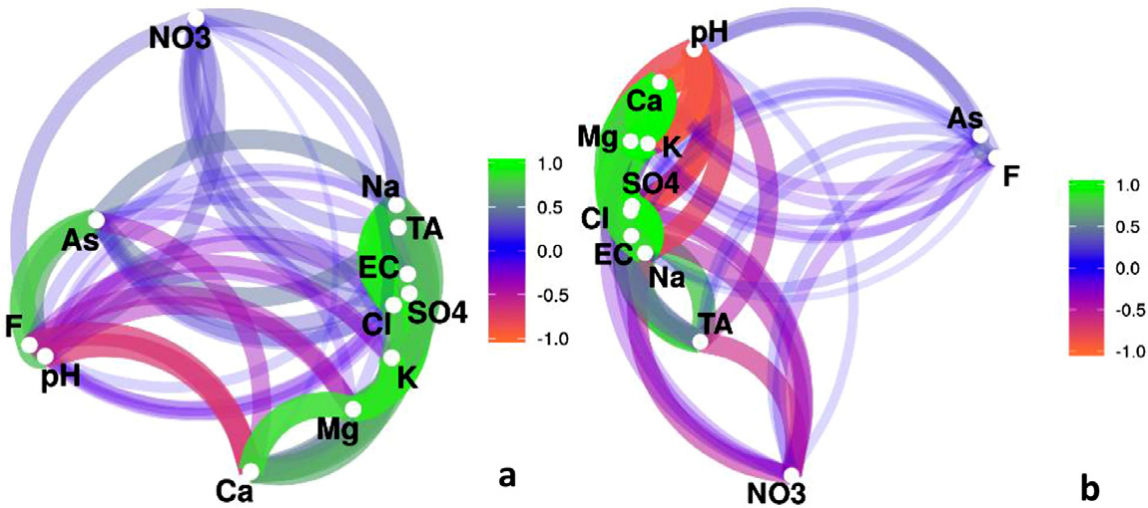
Pearson's correlation coefficient network diagram for (a) shallow piezometers and (b) deep piezometers. Shallow groundwater samples (*n* = 67) and deep groundwater samples (*n* = 48) were recorded during 2018 and 2019 (summer and spring seasons).

Fig. 2 shows the Pearson's correlation coefficient network diagram performed with data from shallow piezometers (a) and deep piezometers (b).

**Fig. 3:**
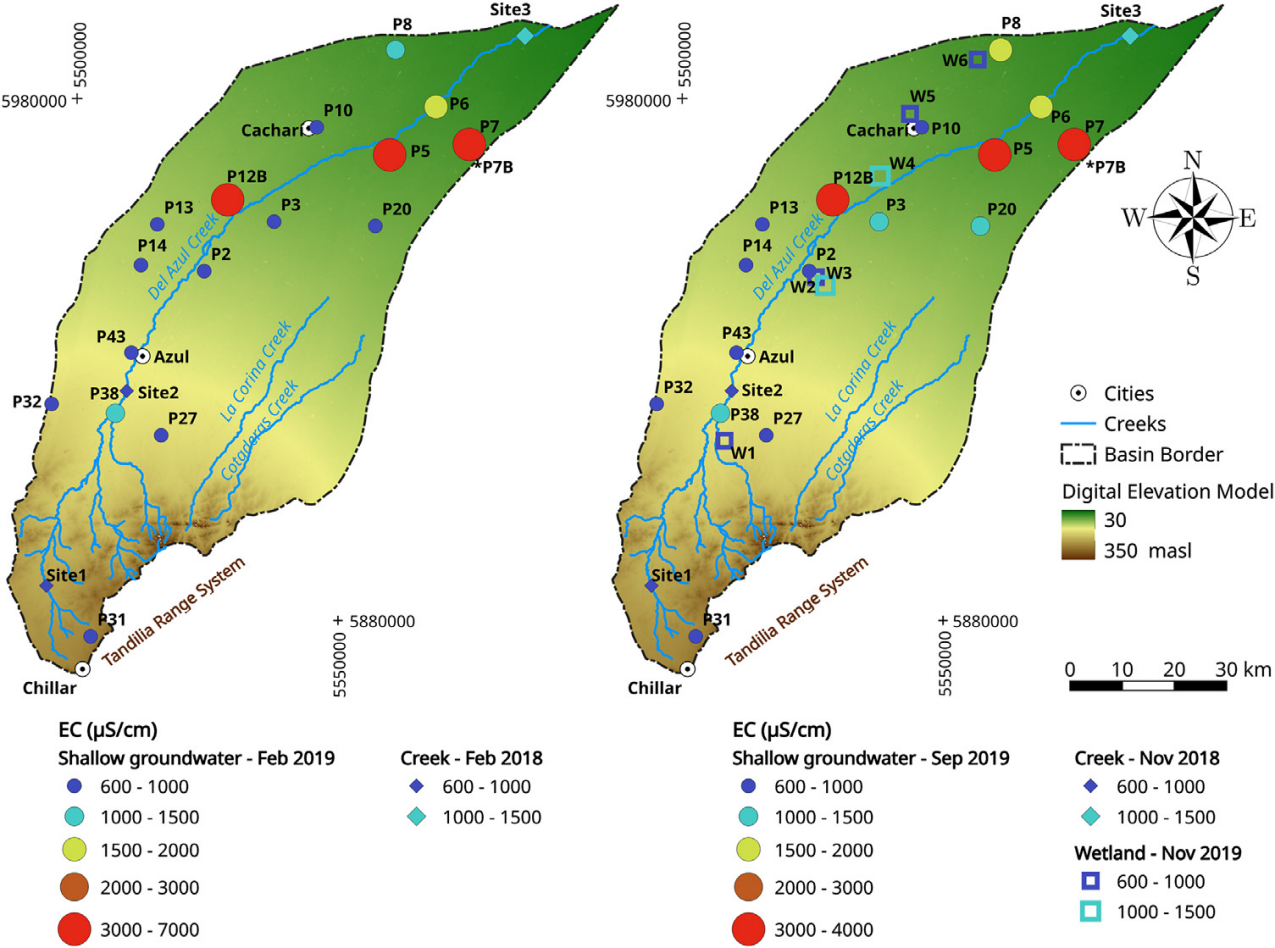
Electrical conductivity (EC) spatial distribution in surface water (creek and wetlands) and groundwater (at 3–10 m depth). Creek EC values were obtained from the sampling campaigns performed in September 2018, whereas the wetlands and groundwater EC were obtained in October 2019.

Fig. 3 shows the spatial surface and shallow groundwater variation in salinity within the Del Azul Creek basin in two opposite hydrological situations (dry summer - February and humid spring - September/November). For the sake of clarity, only shallow groundwater EC values (3–10 m depth) were included in the plots. However, EC from deep boreholes (30 m) follow the same trend.

**Fig. 4:**
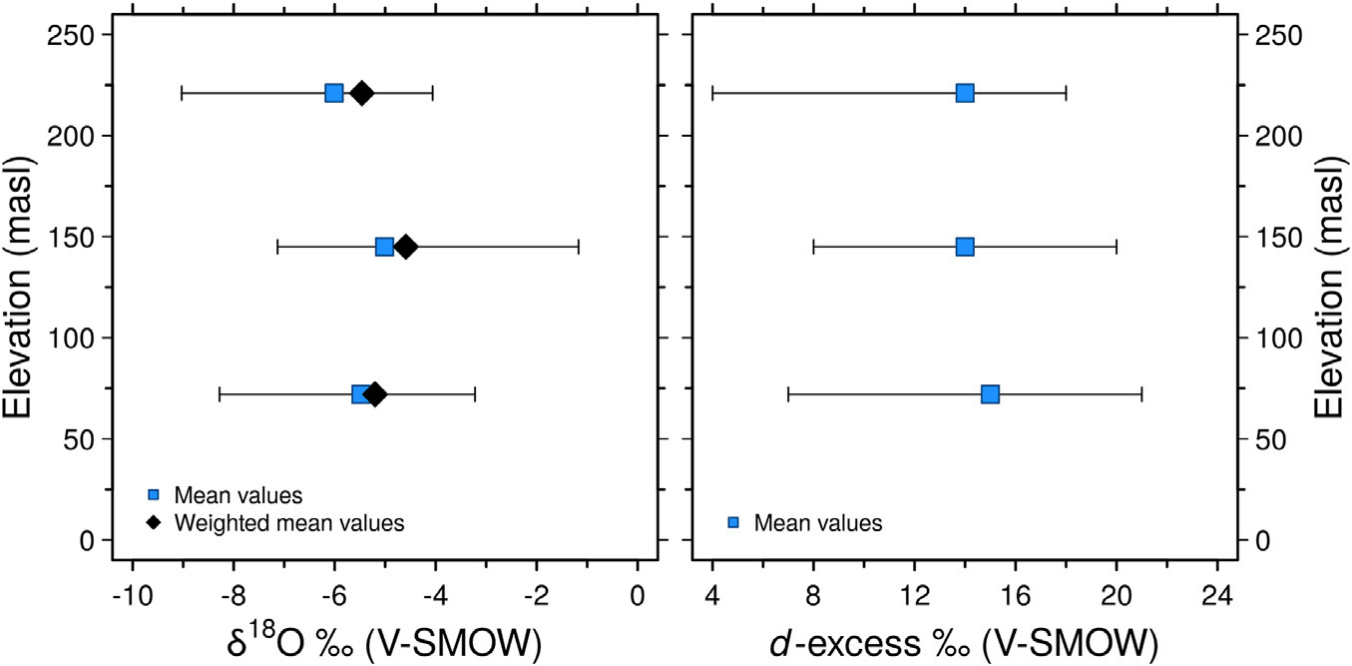
a. Rainfall δ^18^O vs. elevation (masl). b. Rainfall *d*-excess vs. elevation. The mean values and precipitation weighted means were calculated at each sampling site (*n* = 17) with data recorded from 2011 to 2019. The minimum and maximum values were also included.

Fig. 4 shows the rainfall mean isotopic values and the weighted average composition in the entire monitoring period (2011–2019) at each sampling site (LG-upper basin, IHLLA-middle basin and LM-lower basin).

**Fig. 5:**
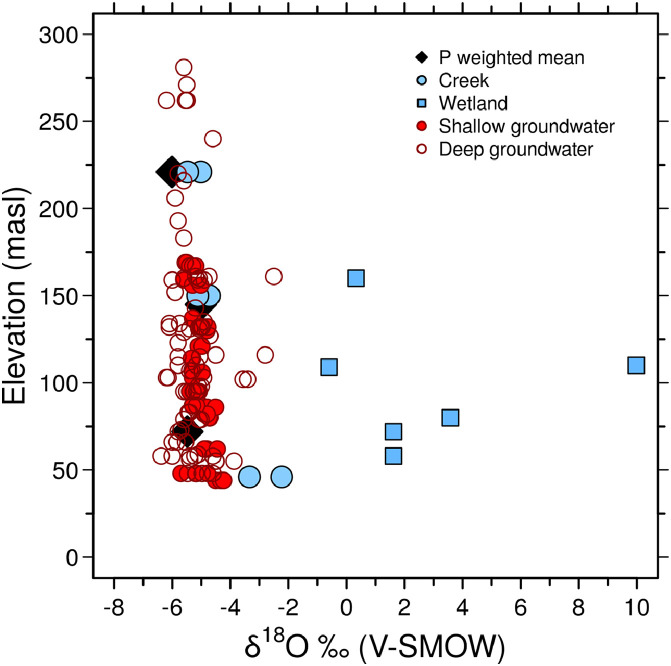
δ^18^O vs. elevation relationship for precipitation (P), surface water (creek and wetlands) and groundwater samples. The precipitation weighted means were calculated at each sampling site (*n* = 17) with data recorded from 2011 to 2019, creek samples (three sampling sites, *n* = 6) were recorded in 2018 (summer and spring seasons), wetlands samples (six sampling sites, *n* = 6) were recorded in 2019 (spring season), shallow groundwater samples (seventeen piezometers, *n* = 67) and deep groundwater samples (twelve piezometers, *n* = 48) were recorded during 2018 and 2019 (summer and spring seasons).

Fig. 5 integrates the δ^18^O contents vs. elevation of the rainwater, creek, wetlands and groundwater in the Del Azul Creek basin.

In addition, a table and a KMZ file were uploaded as supplementary data files to Mendeley repository [7]. The table includes the corresponding information for each sampling site, such as ID, coordinates, elevation values, depths. On the other hand, chemical and isotopic analyses of rainwater, creek, wetlands and groundwater and hydrological data, such as flow rates and piezometric levels, are provided. The KMZ file includes the location of all sampling sites.

## 2 Experimental Design, Materials and Methods

Three integrating wet/dry precipitation collectors and rain gauging stations were installed at different altitudes in the upper (LG), middle (IHLLA) and lower basin (LM), ranging from 221 to 72 masl, respectively. Passive precipitation collectors consisted of a 26.5 cm diameter plastic funnels connected to 20 L plastic containers. Containers with a mineral oil thin-layer were buried underground to avoid secondary evaporation [2,8]. The sampling period was from March 2010 to September 2019. Monthly rainfall amounts were recorded using automated weather stations with remote data transmission at LG and were provided by the Argentina National Weather Service at IHLLA and manually at LM. Although precipitation samples were collected monthly, they were integrated into six-month composite samples for isotopic (*n* = 51) and physico-chemical analyses (*n* = 57).

Three surface water sites were sampled (*n* = 6) along the Del Azul Creek in February 2018 (summer season) and November 2018 (spring season). The superficial flow rates were measured with an OTT MF pro-Water Flow Meter equipment (Site 1) and calculated by rating curves at control sections (Sites 2 and 3). Six physico-chemical analyses and six stable isotope analyses are also available. In addition, six wetlands were sampled (W1 - W6). In this case, six physico-chemical analyses and six stable isotopes analyses for October 2019 (spring season) are also provided in this dataset.

The IHLLA's groundwater monitoring network is composed of 66 piezometers clustered into shallow and deep, but since 2017 only 29 boreholes have been sampled every six months. Shallow piezometers (3–10 m depth) are screened in their last meter, while in the deep piezometers (30 m) screens are located between 25 and 30 m depth. In each groundwater sampling campaign, piezometric levels were also measured. Groundwater samples were collected after purging at least three times the borehole water volume and after EC, pH and water temperature were stabilized [2]. These parameters were measured by a multi parameter probe (Oakton PCSTestr 35) and inside a flow-through cell. One hundred and fifteen physical-chemical analyses for January/February and September/October 2018–2019 and twenty nine stable isotopes analyses for October 2019 are available.

## Declaration of Competing Interest

The authors declare that they have no known competing financial interests or personal relationships which have, or could be perceived to have, influenced the work reported in this article.

## References

[1] INDEC- Instituto Nacional de Estadística y Censos (2010). Censo Nacional de Población Hogares y Viviendas. https://www.indec.gob.ar/indec/web/Nivel4-CensoNacional-3-999-Censo-2010.

[2] Zabala M.E., Gorocito M., Dietrich S., Varni M., Sánchez Murillo R., Manzano M., Ceballos E. (2020). Key hydrological processes in the Del Azul Creek basin, sub-humid Pampean plain. Sci. Total Environ..

[3] APHA (2012). Standard Methods for the Examination of Water and Wastewater.

[4] Coleman M.L., Sheperd T.J., Durham J.J., Rouse J.E., Moore F.R. (1982). A rapid and precise technique for reduction of water with zinc for hydrogen isotope analysis. Anal. Chem..

[5] Panarello H.O., Parica C.A. (1984). Isótopos del oxígeno en hidrogeología e hidrología. Primeros valores en aguas de lluvia de Buenos Aires. Rev. Asoc. Geol. Argent..

[6] Lis G., Wassenaar L.I., Hendry M.J. (2008). High-precision laser spectroscopy D/H and ^18^O/^16^O measurements of microliter natural water samples. Anal. Chem..

[7] Zabala M.E., Sánchez Murillo R., Dietrich S., Gorocito M., Vives L., Manzano M., Varni M. (2020).

[8] Zabala M.E., Manzano M., Vives L. (2015). The origin of groundwater composition in the Pampeano Aquifer underlying the Del Azul Creek basin, Argentina. Sci. Total Environ..

[9] Zabala M.E., Manzano M., Vives L. (2016). Assessment of processes controlling the regional distribution of fluoride and arsenic in groundwater of the Pampeano Aquifer in the Del Azul Creek basin (Argentina). J. Hydrol. (Amst).

